# The Frequency of Alcohol Use in Iranian Urban Population: The Results of a National Network Scale Up Survey

**DOI:** 10.15171/ijhpm.2016.103

**Published:** 2016-08-17

**Authors:** Ali Nikfarjam, Saiedeh Hajimaghsoudi, Azam Rastegari, Ali Akbar Haghdoost, Abbas Ali Nasehi, Nadereh Memaryan, Terme Tarjoman, Mohammad Reza Baneshi

**Affiliations:** ^1^Emergency Medical Center, Ministry of Health and Medical Education, Tehran, Iran.; ^2^HIV/STI Surveillance Research Center, and WHO Collaborating Center for HIV Surveillance, Institute for Futures Studies in Health, Kerman University of Medical Sciences, Kerman, Iran.; ^3^Iran Helal Institute of Applied-Science and Technology, Tehran, Iran.; ^4^Faculty of Behavioral Sciences and Mental Health, Iran University of Medical Sciences, Tehran, Iran.; ^5^Department of Community Medicine, Tehran Medical Branch, Islamic Azad University, Tehran, Iran.; ^6^Modeling in Health Research Center, Institute for Futures Studies in Health, Kerman University of Medical Sciences, Kerman, Iran.

**Keywords:** Alcohol, Abuse, Network Scale Up (NSU), Iran

## Abstract

**Background:** In Islamic countries alcohol consumption is considered as against religious values. Therefore, estimation of frequency of alcohol consumptions using direct methods is prone to different biases. In this study, we indirectly estimated the frequency of alcohol use in Iran, in network of a representative sample using network scale up (NSU) method.

**Methods:** In a national survey, about 400 participants aged above 18 at each province, around 12 000 in total, were recruited. In a gender-match face to face interview, respondents were asked about the number of those who used alcohol (even one episode) in previous year in their active social network, classified by age and gender. The results were corrected for the level of visibility of alcohol consumption. ****

**Results:** The relative frequency of alcohol use at least once in previous year, among general population aged above 15, was estimated at 2.31% (95% CI: 2.12%, 2.53%). The relative frequency among males was about 8 times higher than females (4.13% versus 0.56%). The relative frequency among those aged 18 to 30 was 3 times higher than those aged above 30 (3.97% versus 1.36%). The relative frequency among male aged 18 to 30 was about 7%.

**Conclusion:** It seems that the NSU is a feasible method to monitor the relative frequency of alcohol use in Iran, and possibly in countries with similar culture. Alcohol use was lower than non-Muslim countries, however, its relative frequency, in particular in young males, was noticeable.

## Introduction

Iran, a Middle East country with an Islamic culture, has a population of about 75 millions. Iran has a young population where around one-third (35%) of the population is aged 15 to 29 years old.^1^

In recent years, alcohol consumption has become an important concern, as it may increase the risk of alcohol-related disorders,^2,3^ and unsafe sexual contacts globally.^4,5^ Therefore, monitoring the frequency of alcohol use is a priority to health professionals for policy-making and planning.^1,6^ This issue is more important in Iran which based on its dominant culture alcohol use is not an acceptable practice, and the trade of alcohol is illegal.

In order to get an impression about the frequency of alcohol use at province and national level, we systematically reviewed all publications in Iran (under preparation). In total 147 manuscripts were included. However, majority of them focused on a specific subpopulation (such as students). Furthermore, prevalence (either life time or last year) for only 17 provinces were available. In total, only 11 manuscripts reported last year prevalence of which the subpopulation of 9 ones was students.

The main reasons why results of our systematic review do not provide a representative national estimate were as follow:

1. The target group in majority of these studies was only students in high schools or universities. In addition, the past year prevalence of alcohol use even in this group ranged from 2.04% (among students in Hormozgan province)^7^ to 68.8% (among adolescent and high school students in Tehran).^8^ It has been shown that the alcohol consumption was related to factors such as: age and living place.^9-11^ As students are usually young and part of them live in dormitories without supervision of their families, such estimates are not representative of the whole community.
2. Some of these estimations were extracted from the results of studies that were designed for some other objectives. For example, in a population-based cohort in north east of Iran, to check main risk factors of cancers, the life time prevalence of alcohol use was 2% in cases (N = 300) and also in matched controls (N = 571).^12^ As alcohol use in Iran is not that prevalent, a larger sample size is required to get more precise estimates. With 95% confidence, a sample size of about 4000 is required to provide an accurate estimate for a prevalence of 2%, therefore, we believe that such estimates might not be the most valid estimates even for that geographical zone.
3. The results of life time prevalence of alcohol use among general population in some of provinces were available with a wide range of variation 2% in Gonabad city (Golestan province),^12^ to 26.6% in Shiraz city (Fars province).^13^ Also no data is available for 14 provinces. Therefore, extrapolation of figures to other provinces to get a figure for the whole community is difficult. In addition, it is difficult to identify hot spots across the country.
4. From the methodological point of view, data were collected differently; mainly used direct methods such as interview or self-administered questionnaire. Such methods are prone to information bias because social barriers for alcohol consumption in Iran.^14,15^

Because of these limitations, the Ministry of Health and Medical Education (MoHME) of Iran was convinced to estimate the frequency and the pattern of alcohol use using an indirect method in a national wide study. Among the list of indirect methods, network scale up (NSU) was selected as the most feasible design to address to this need after a long discussion and evaluation in national level.

Briefly, in the NSU, respondents are asked to describe the prevalence of risky behaviors in their social network rather than themselves. The feasibility and validity of NSU method has been examined in several studies; for example in the estimation of the prevalence of HIV,^16^ rape and homelessness and also heroin users in the United States,^17^ key affected populations (KAPs) in Ukraine,^18^ men who have sex with men (MSMs) in Japan,^19^ and female sex workers in Kenya.

On top of the international experiences, and before this national study, we estimated the frequency of drug use and sexual risky behaviors in one of big cities in Iran using NSU method.^20^ In addition, we estimated the size of social network of Iranian population (C) which is one of key input parameters for NSU.^21^

Based on the above explanation, we designed a national survey to explore the frequency of different risky behaviors in Iran using NSU technique. In this paper, we presented our main findings about the frequency of alcohol use in Iran, at the province and national level.

## Methods

### Sample Size and Data Collection

This cross-sectional study has been conducted in Iran in 2012 which is composed of 31 provinces. Multistage sampling technique was used to select a random sample from above 18 years old of urban people. The sample size in each province was around 400, except in Tehran which was 1000. In each province, around 75% of samples were selected from the capital and the remaining from one or two large cities.

To select the samples, each city was stratified to three zones based on social and economic class. From each stratum, two to four public locations (from main streets and square) were selected randomly. For data collection, street-based sampling was used because our previous experience in comparison of different interview methods (street-based, home-based, and telephone-based), showed that Iranian people reply to sensitive questions more accurately through street-based approach.^22^

Only sole pedestrians were approached by a same sex interviewer. To get a more representative sample, maximum variation rule was used to recruit samples with maximum heterogeneity in their demographic variables (ie, clothing, age, gender, education level, etc). The interviewers explained the main objectives of the study to the pedestrians ensuring the anonymity and confidentiality of their answers. Missing rate was less than 5%. Therefore, subjects with complete data were analyzed.

### Network Scale Up Analysis

The assumption behind NSU is that on the overall people’s network represents the target population fairly.^18^ The NSU requires the estimation of average network size of the population (shown by C). We calculated the average network size of Iranian at 308.^21^

The basic formula for calculating personal network size is:

e/t = m/c

Here, *m* is the average number of people belonging to a reference subpopulation who were known by our samples, *C* is the active network size, *e* is the size of the reference subpopulation, and *t* is the size of the total population. Combining information of several reference groups, the above formula can be modified to get better estimates:

$$c_{i}=t\frac{\sum_{i}^{}m_{ij}}{\sum_{i}^{}e_{j}}$$

We simply asked respondents how many people they knew (shown by *m*) who consumed alcohol at least once in previous year, stratified by gender, and three age groups (<18, 18-30, >30). According to the usual practice ‘know’ was defined as mutual recognition of each other by sign or name (may be contacted) and in the past two year have had contact either in person, face-to-face, phone, or by email.^23,24^

Before data analysis the data were cleaned extensively. Responses above 30 were rounded to 30. In addition, if the difference between total *m* and summation of male *m* and female *m* was more than 2, that respondent was removed from all the analysis. Also, as three age groups were defined, if the difference between total *m* and summation of replies in 3 age groups were more than 3 that respondent was removed.

Estimation of frequency of alcohol user in the first step was based on below formula:

$${\overset{⌢}{e}}_{j}=t\frac{\sum_{i}^{}m_{i}}{\sum_{i}^{}c_{i}}$$

Where: *T* is total population, *M*_i_ is number of people in active network of respondent size as alcohol user, and is active network size of Iranian population.

After that this value (${\overset{⌢}{e}}_{j}$) was divided by transparency factor.

### Adjustment for Transparency

One potential limitation of NSU studies is transmission error: some of the members of a respondent’s network might have used alcohol, but the respondents might not be aware of that. Visibility factor (VF) has been defined as proportion of alters (ie, members of network) of an ego (ie, person who practice risky behavior) who are aware of his risky behavior. In a recent study, we estimated VF for injection drug users (IDUs) at 0.54. This means that size estimations derived by NSU study, should nearly be doubled to correct for visibility of this behavior in the society.

Since, the visibility of alcohol use was a point of concern, the results of this analysis were corrected using 0.54 as the visibility coefficient (adopted from Brazil experience).^25^ In other words, it was assumed that only around half of the social network of each Iranian people has knowledge about their alcohol consumption behaviors.

### Assessment of Factors Influence Replies

Negative binomial regression model was fitted to investigate the impact of demographic characteristics of respondents on their response. This model is similar to Poisson regression model but is used when mean and variance are not the same. This model allows for over dispersion in data, a phenomena common in count data sets. In addition, the number of alcohol users in previous year, and relative frequencies were computed for the whole country (stratified by age group and gender) and in each provinces. Having used these calculations, provinces were classified into four groups based on quartiles.

### Uncertainty Limits

To provide uncertainty level for estimates, we applied Monte Carlo technique. We assumed that *m* (the number of alcohol users in social network of each subject), and visibility coefficient follows Poisson and uniform distributions, respectively. In 1000 iteration, *m* took a random number from a Poisson distribution with mean and variance equal to the average of *m* in all subjects. Uniform distribution in a range between 0.44 and 0.64 was used to generate a random value for the visibility coefficient. The lower and upper bounds of 95% uncertainty levels were set at 2.50 and 97.50 percentiles in 1000 iterations.

All analyses were done using SPSS and Microsoft Excel software. The maps were created using Arc Map version 9.3 software.

## Results

In total 12 293 samples, including 6213 males (50.54%) and 6080 females (49.46%) were recruited. The age of participants ranged 18 to 87 years; mean ± standard deviation (SD) age was 32.78 ± 11.24. Nearly 61% of sample was married, and 44% of subjects had college education. Comparing age and gender distribution of our sample with that of the Iranian general population, no significant difference was seen.

Nearly half of respondents did not know anyone who used alcohol in the past year in their networks. The mean ± standard error (SE) of the number of alcohol users in the social network of males and females were 3.94 ± 0.56 and 2.30 ± 0.36, respectively (Table 1). Males knew higher mean number of people who consumed alcohol than female participants (incidence rate ratio [IRR] = 1.71), giving a *P* < .001. The men expected number of people who had consumed alcohol knew by male and female was 3.94 and 2.30, respectively, giving IRR of 1.71.

**Table 1:** The Association of Demographic Characteristics of Subjects on Number of Reported Alcohol Users Known, Derived Applying Negative Binomial Regression

**Variable**	**Mean (SE)**	**IRR (95% CI)**	***P*** ** Value**
Gender			
Female	2.30 (0.35)	REF	
Male	3.94 (0.56)	1.71 (1.39, 2.11)	<.001
Age			
18-30 years	3.56 (0.49)	REF	
>30 years	2.55 (0.39)	0.71 (0.61, 0.83)	<.001
Marital status			
Married	2.63 (0.32)	REF	
Divorce/widow	3.04 (0.70)	1.16 (0.84, 1.60)	.370
Single	3.41 (0.39)	1.30 (1.12, 1.51)	.001
Education			
<12 years	2.53 (0.32)	REF	
12-16 years	3.11 (0.45)	1.22 (1.10, 1.36)	<.001
>16 years	3.46 (0.57)	1.37 (1.13, 1.65)	.001

Abbreviations: SE, Standard error; IRR, incidence rate ratio.

On the other hand, relative to younger respondents, older respondents knew lower mean number of people who used alcohol (IRR = 0.71, *P* < .001). Mean ± SE known by those aged above 30 and those aged 18 to 30 were 2.55 ± 0.38 and 3.56 ± 0.49, respectively. In addition, we found a positive association between education level and the number of known alcohol users in the social network of subjects (Table 1).

The estimated number of those who used alcohol at least once in the last year was 1 300 858 (95% CI: 1 195 530-1 426 513; Table 2). This was corresponded to a relative frequency of 2.31% (95% CI: 2.12%, 2.53%). The ratio of number of males to females who used alcohol in the past year was about 7.50 (1 168 624 males [95% CI: 1 072 879-1 282 850] versus 158 546 females [95% CI: 144 685-174 916]). The relative frequency in males and females were 4.13% (95% CI: 3.79%, 4.53%) and 0.56% (95% CI: 0.52%, 0.62%), respectively.

**Table 2:** Estimation of Annual Frequency and Prevalence of Alcohol Use Among Iranian General Urban Population^a^

**Population**	**Frequency (CI)**	**Relative Frequency % (CI)** ^b^
Total	1 300 858 (1 195 530-1 426 513)	2.31 (2.12-2.53)
Male	1 168 624 (1 072 879-1 282 850)	4.13 (3.79-4.53)
Female	158 546 (144 685-174 916)	0.56 (0.52-0.62)
Age <18	40 421 (36 038-45 445)	1.57 (1.40-1.77)
Age 18 to 30	856 863 (786 778-940 516)	3.97 (3.65-4.36)
Age >30	440 786 (403 632-484 364)	1.36 (1.25-1.50)

^a^Summation for subgroups dose not exactly match the total estimate, because of our data cleaning protocol. ^b^To calculate these CIs, corresponding confidence in frequency column are divided by each group’s population.

The estimated number of users in aged between 15 and 18, 18-30 and >30 years old people were 40 421, 856 863 and 440 786, respectively. The relative frequency of alcohol consumption among those aged 18 to 30, was 3 times higher than those aged above 30 (3.97% versus 1.36%). Corresponding figure in comparison with those aged less than 18 was about 2.50 (3.97% versus 1.57%).

We have seen that the relative frequency among males and among those aged 18 to 30 was much higher than other groups. As explained in methods section, we have not asked respondents to cross-classify their response by both age and gender. Therefore, considering a 3 by 2 table (corresponded to 3 age categories and 2 gender level), only marginal replies were available. To get a figure for males aged 18 to 30, we used a ratio of 7.50 to divide total number of alcohol users in this age group to male and female users. We estimated than 756 056 out of 856 863 were males. This means that past year prevalence of alcohol use among males aged 18 to 30 was about 7%.

Figure  presents the geographical distribution of alcohol use across the country. A cluster of higher users was observed in the center of Iran and west of Iran, but a clear geographical pattern was not observed.

**Figure:**
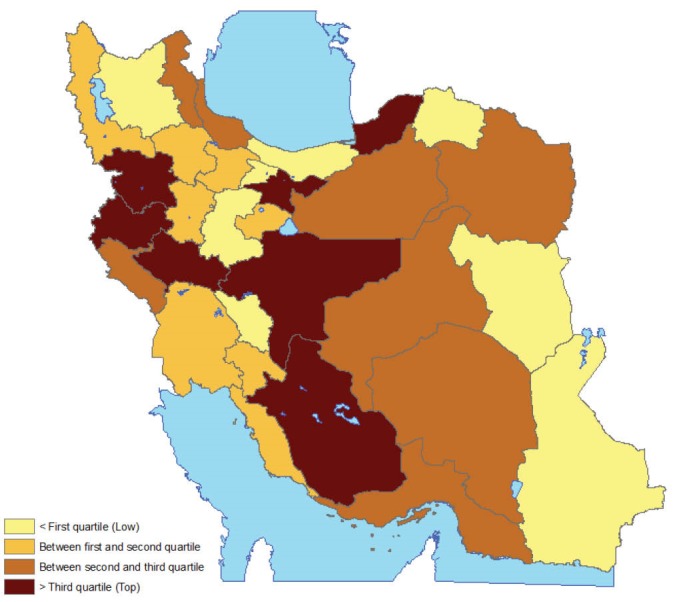
Geographical Distribution of Alcohol Abuse in Iran.

## Discussion

Based on our best knowledge, this is the first study that used NSU method to estimate the frequency of alcohol consumption in the general population in the Middle East region at national level. In this study, we estimated the annual frequency of alcohol consumption in Iranian general population classified by gender and age group. We checked distribution of age and gender of our sample, this distribution was same as urban Iranian population.

In our extensive search, we did not find any comparable study that focuses on the whole population. In a few general population studies the frequency of consumption were assessed only in a limited geographical zone in a specific age group only using direct methods which are usually under-estimate such a culturally sensitive frequency.

Overall, relative frequency of alcohol use in the general population was estimated at 2.3%. In accordance to the usual practice, we rounded the high values to 30.^16^ Considering the average network size at 308, this means that we assumed that at most 10% of network of each respondent is engaged in risky behavior. As a sensitivity analysis, we applied 50 as the ceil value. This resulted in a prevalence of 1.9%. In one of the World Health Organization (WHO) reports, the prevalence of abstainers in the last 12 months among those aged >15 was estimated at 95.8%, corresponded to a past year prevalence of alcohol use of 4.2%.^26^ We could not find methodological details of WHO report; therefore, comparison of results is not simple. However, we believe that two percentage points difference is not noticeable. This is due to the fact that alcohol drink in Iran is illegal and invisible. Besides that, results of other studies implemented in Iran are hugely different.

Based on our systematic review, life time prevalence ranged from 2% in Golestan,^12^ to 26.6% in Shiraz city.^13^ However, the target population in Golestan study were rural and urban subjects aged >35. Target population in Shiraz study was urban subjects aged >15. Furthermore, methods of data collection in these two studies were interview, and household semi-structured interview. On the other hand, in our study, more than 12 000 urban respondents reported past year prevalence of alcohol use in their network.

Comparing our results with western countries, we did not find any figure that represents the annual frequency of alcohol in the whole general population. In addition, definition of alcohol use in our study and Western studies were not the same. In Ireland (2005) 77% of all 18–64-year-olds reporting they had consumed alcohol in the previous year, and 34% usually consuming at least five drinks per drinking occasion.^27,28^ In Scotland, 39% of men and 31% of women consumes more than twice the recommended daily maximum units on their heaviest drinking day each week.^27,28^ The prevalence of alcohol consumption among Korean adult population was reported at 79%.^29^ These together suggest remarkable difference between Iran and western countries.

In terms of gender, we observed that male respondents were more likely to know alcohol users. Alcohol use among males was much more prevalent than females (3.1% versus 0.4%). Several studies have shown that alcohol use among males is much higher than females.^3,30^ This might be due to the fact that in Islamic countries that the alcohol consumption is illegal for all but stigma against women is much stronger.^31^ In addition, males have reported greater affirmative expectancies form alcohol and they drink alcohol more likely to as a way of coping mechanism.^32^

In terms of impact of age on alcohol use, the prevalence among young generation was higher than other age groups. Almost 856 900 people in the age range of 18 to 30 had used alcohol at least once in the recent year, which shows past year prevalence of alcohol use 3.97% among Iranian young generation. Considering the fact that the alcohol use is much more uncommon in the females, and with the concept that almost half those formed this age group are females, the past year prevalence of alcohol use among males 18 to 30 years old is about 7%. Also alcohol consumption seems prevalent among male, specific strategies for this group should be dispensed by policy-makers. Because of the destructive impact of alcohol on public health, the policy-makers must be designed some plans to reduce harmful effects. They must notice that preventive intervention much better than treatment.

Our figure for males aged 18 to 30 (ie, 7%) is more or less the same as Shokoohi et al study in which, using NSU method, past year prevalence of alcohol use among Kermanian males aged 18 to 45 was reported at 6.8%.^33^ Association between age and alcohol use was confirmed in lots of other studies.^9-11^ Other studies focused on the young generation was mainly performed among high school or university students, which are not representative of the whole population. Among published manuscript, the minimum and maximum life time prevalence among male students was estimated at 11%^34^ and 33%^35^ among Kerman and Shiraz students, respectively. Both of these studies used self-administered questionnaires.

Our study had several limitations. At each province, we only recruited samples from province center and one or two major cities. This was the first national NSU study in Iran, and from practical point of view, it was extremely difficult to cover the rural areas. Therefore, the results cannot be generalized to rural population.

Additionally, the social transmission has not been calculated as we had no access to a random sample of alcohol users. We, therefore, adopted the Brazilian figure, 0.54. However, social norms, religious values, and rules, affect the level of visibility.^36^ Even in a country, the visibility in all provinces is not necessarily the same. Our experience in estimation of visibility of being injected drug user and female sex worker, reveals that the visibility of risky behaviors among women is significantly lower than males.^37^ Further studies should be designed to estimate this correction factor for Iranian population, stratified by gender and age group, and possibly province.

The next issue is that street-based method might not necessarily provide the best possible representative sample. This is because some subgroups such as old and sick people might not be selected. However, in NSU, respondents reply on behalf of their network but not themselves. Therefore, such subgroups were indirectly involved in the study.

One more issue is that the study designed to provide a robust figure at national level. Although our sample size at each province was not large enough, and therefore, province level estimates might not be robust, we believe that level of bias in size estimation in all provinces is more or less the same and therefore, this does not seriously affect our risk zones.

Other frequently used size estimation methods include capture-recapture and multiplier. However, it was not possible to implement them in Iran’s setting. Therefore, we were not able to triangulate and cross validate our results.

Besides limitation noted, this was the first national level NSU study has been implemented in the Middle East region. Recruiting a large sample and implementing the study in all provinces are other main strengths of our study.

Studies implemented so far does not follow a standard and validated approach. On the other hand, it seems that the NSU is a feasible method for evaluation of the size of the hidden groups such as alcohol use in Iran setting. Current study shows that alcohol use in Iran was lower than non-Muslim countries. However, its relative frequency is noticeable especially among the youth. Therefore, a comprehensive national strategy needs to address and prioritize the full range of harmful outcomes when making high-level decisions about policy priorities and investment.^19^

## Acknowledgments

This work has been granted by the Iranian Ministry of Health and Medical Education (MoHME), Tehran, Iran.

## Authors’ contributions

AN, AAH, AAN, NM, TT, and MRB designed the study. AR and SH managed and monitored the data collection and cleaning process. AAH, AR, SM, and MRB analyzed the data. The first draft of the manuscript was prepared by AN, AR, SH, AAH, and MRB. All authors have read and approved the final manuscript.

## Competing interests

All of the authors are independent researchers without any conflict of interest except for AAN and AN, who were the heads of the Department of Addiction, Deputy of Mental and Social Health, Health Deputy of MoHME and NM and TT, members of the Department of Addiction, Deputy of Mental and Social Health, Health Deputy of MoHME. These authors indirectly involved with the concept of alcohol. All authors have read and approved the final manuscript.

## Ethical issues

The proposal of this survey was approved by ethical committee of Kerman University of Medical Sciences, Kerman, Iran (ethical committee code: 163/90/KA).

## Authors’ affiliations

^1^Emergency Medical Center, Ministry of Health and Medical Education, Tehran, Iran. ^2^HIV/STI Surveillance Research Center, and WHO Collaborating Center for HIV Surveillance, Institute for Futures Studies in Health, Kerman University of Medical Sciences, Kerman, Iran. ^3^Iran Helal Institute of Applied-Science and Technology, Tehran, Iran. ^4^Faculty of Behavioral Sciences and Mental Health, Iran University of Medical Sciences, Tehran, Iran. ^5^Department of Community Medicine, Tehran Medical Branch, Islamic Azad University, Tehran, Iran. ^6^Modeling in Health Research Center, Institute for Futures Studies in Health, Kerman University of Medical Sciences, Kerman, Iran.

## Key messages

**Implications for policy makers**

- Our study provides as updated prevalence for alcohol use.
- Alcohol user common among young male.
- Hot zones in term of alcohol use are defined.

**Implications for public**

Alcohol consumption may increase the risk of alcohol-related disorders, effects on public health by impact on different disease such as hypertension, cancers, cardiovascular disease Almost 856 900 people in the age range of 18 to 30 had used alcohol at least once in the recent year, which shows prevalence 3.97% among Iranian young generation. Considering the fact that the alcohol use is much more uncommon in the females, and with the concept that almost half those formed this age group are females, the annual prevalence among males 18 to 30 years old is about 7%. Also alcohol consumption seems prevalent among male, specific strategies for this group should be dispensed by policy-makers. Because of the destructive impact of alcohol on public health, the policy-makers must be designed some plans to reduce harmful effects. They must notice that preventive intervention much better than treatment.
